# Witness-based nonlinear detection of quantum entanglement

**DOI:** 10.1016/j.isci.2025.112174

**Published:** 2025-03-10

**Authors:** Yiding Wang, Tinggui Zhang, Xiaofen Huang, Shao-Ming Fei

**Affiliations:** 1 School of Mathematics and Statistics, Hainan Normal University, Haikou 571158, China; 2 School of Mathematical Sciences, Capital Normal University, Beijing 100048, China

**Keywords:** Physics methods, Theoretical physics, Quantum theory

## Abstract

We present a nonlinear entanglement detection strategy that detects entanglement that the linear detection strategy fails. We show that when the nonlinear entanglement detection strategy fails to detect the entanglement of an entangled state with two copies, it may succeed with three or more copies. Based on our strategy, a witness combined with a suitable quantum mechanical observable may detect the entanglement that cannot be detected by the witness alone. Moreover, our strategy can also be applied to detect multipartite entanglement by using the witnesses for bipartite systems, as well as to entanglement concentrations.

## Highlights

• A nonlinear entanglement detection strategy is presented
• This strategy is generalized by adopting multi-copies
• Nonlinear witness combined with a positive semidefinite observable can detect entanglement
• This strategy can be applied to entanglement concentrations

## Graphical abstract

**Figure:**
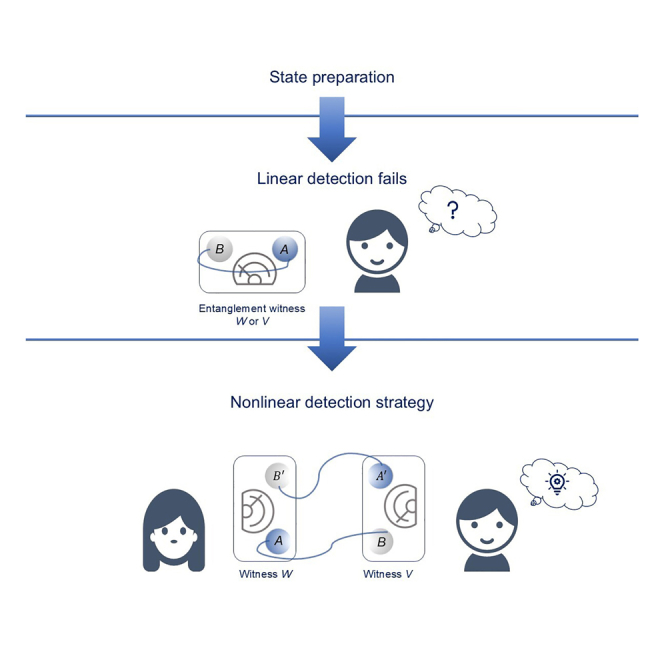

## Introduction

One of the most distinctive features of quantum mechanics is the quantum correlations. The quantum entanglement^1^ is the most extensively studied quantum correlation, promising advantages over the classical correlations in many quantum information tasks such as quantum communications,^2^^,^^3^^,^^4^ quantum computing,^5^^,^^6^^,^^7^ quantum simulation,^8^ and quantum cryptography.^9^^,^^10^^,^^11^

For the study of quantum entanglement, a basic issue is to determine whether a state is entangled or not. However, this problem is typically N-P hard.^12^ Practical entanglement detection not only needs to ensure efficiency, that is to say use far fewer local measurements than those needed for state tomography, but also has adequate detection power.^13^^,^^14^ The entanglement witnesses^15^ play important roles in experimental detection of quantum entanglement for unknown quantum states.^16^^,^^17^ Entanglement witnesses are observables that are nonnegative for all separable states and negative for some entangled states and have been widely studied in recent years.^18^^,^^19^^,^^20^^,^^21^^,^^22^^,^^23^^,^^24^^,^^25^^,^^26^^,^^27^^,^^28^^,^^29^^,^^30^^,^^31^^,^^32^^,^^33^^,^^34^^,^^35^^,^^36^^,^^37^^,^^38^^,^^39^^,^^40^^,^^41^^,^^42^ In 2010, Jurkowski et al.^18^ show that each entanglement witness detecting the entanglement of a given bipartite state provides an estimation of the concurrence of this state. Hou et al.^19^ provide two methods of constructing entanglement witnesses that detect entanglement that cannot be detected by the positive partial transpose (PPT) criterion and the realignment criterion. A series of new ultrafine entanglement witnesses have been proposed,^27^^,^^28^ which can detect entangled states that the original entanglement witnesses cannot. In 2021, Sen et al.^31^ obtain an upper bound on the entanglement witness function in the measurement-device-independent entanglement witness scenario. General and robust device-independent witnesses have been explored,^32^ which can be applied to identify entanglement in various arbitrary finite dimensional multipartite quantum systems, based merely on bipartite Bell inequalities. Shi^38^ presents a method to obtain the lower bounds of concurrence, entanglement of formation, and geometrical entanglement measure based on entanglement witnesses. The authors extend the entanglement-certification toolbox from correlations in mutually unbiased bases to arbitrary bases, even without requiring aligned reference frames.^41^ Sun et al.^42^ present an approach to estimate the operational distinguishability between an entangled state and any separable state directly from the measurement of an entanglement witness.

Nonlinear entanglement detection is currently the research focus,^43^^,^^44^^,^^45^^,^^46^^,^^47^ which can outperform linear counterparts.^48^^,^^49^^,^^50^^,^^51^ Liu et al. prove that if multi-copy joint measurements are allowed, the effectiveness of entanglement detection can be exponentially improved.^44^ Yamasaki et al.^45^ characterize the genuine multipartite entanglement in the paradigm of multiple copies and conjecture a strict hierarchy of activatable states. Rico et al. introduce a systematic method for nonlinear entanglement detection based on trace polynomial inequalities.^47^ The authors provide a method to construct nonlinear entanglement witnesses,^50^ which improve on linear entanglement witnesses in the sense that each non-linear entanglement witness detects more entangled states than its linear counterpart. Moreover, a nonlinear improvement of any entanglement witness for 2×d quantum systems was introduced.^51^

In fact, any entangled state can be detected by some proper entanglement witnesses; however, the construction of witnesses is not a straightforward work and relies on the specific structure of the state to be detected. Our motivation here is not to search for or construct new entangled witnesses, but to better utilize the existing entangled witnesses to improve their ability of entanglement detection. That is to say, given a set of entanglement witnesses, the question is if they can be still employed in a nonlinear strategy to detect entanglement in multiple copies of an unknown state, when the linear strategy fails. Our answer is positive. We prove that there are indeed witnesses for which the nonlinear strategy detects a different set of entangled states, while the linear counterparts fail to detect (Observation 1 and Observation 2). Moreover, by combining witnesses with a positive semidefinite operator, we show that there exist pairs of states and witnesses such that the linear detection fails, but the nonlinear detection succeeds. Furthermore, the entanglement detection ability can be improved by adjusting the positive semidefinite operators (Observation 3 and Example 4). Our strategy may suggest a new perspective for detecting tripartite entangled states: using bipartite witnesses to measure two copies of the tripartite state (Observation 4 and Example 5). The rest of this paper is organized as follows. In the second section, we provide our nonlinear detection strategy and the related applications. We summarize and discuss our conclusions in the last section.

## Results

### Nonlinear entanglement detection

An *n*-partite quantum state *ρ* is said to be separable if it can be expressed as a convex combination of product states,

$$\rho =\sum_{i}p_{i}\rho_{i}^{1}⊗\rho_{i}^{2}⊗\ldots ⊗\rho_{i}^{n}\text{,}$$

where $0\leq p_{i}\leq 1$, $\sum_{i}\;p_{i}=1$, and $\rho_{i}^{k}$ are the density matrices of the *k*th subsystem. Otherwise, *ρ* is an entangled state. The entanglement witnesses provide powerful tools in detecting the entanglement of unknown quantum states. An entanglement witness is an observable *W* such that $\text{Tr}\left(W\rho_{ent}\right)<0$ for some entangled states $\rho_{ent}$ and $\text{Tr}\left(W\rho_{sep}\right)\geq 0$ for all separable states $\rho_{sep}$. Therefore, *W* gives rise to a hyperplane that separates the entangled states detected by *W* from the convex set of separable states and the entangled states not detected by *W*. Such entanglement detection is called linear detection.

Nonlinear entanglement detection is given by witnesses based on nonlinear improvement of the linear ones,^48^ or by adopting multi-copy scenarios.^45^^,^^47^ Recently, the authors introduced the nonlinear entanglement witness by using tensor product of linear counterparts,^47^

$$W=W_{1}⊗W_{2}⊗\ldots ⊗W_{n}$$

such that $\text{Tr}\left(W\rho_{ent}^{⊗k}\right)<0$ for some entangled states $\rho_{ent}$, while $\text{Tr}\left(W\rho_{sep}^{⊗k}\right)\geq 0$ for all separable states, where *k* depends on the local dimension and *n*. It is shown that there are two bipartite witnesses *W* and *V* and a bipartite entangled state $\rho_{AB}$ such that $\text{Tr}\left(W\rho_{AB}\right)\geq 0$ and $\text{Tr}\left(V\rho_{AB}\right)\geq 0$, but $\text{Tr}\left(W_{AA^{\prime }}⊗V_{BB^{\prime }}\rho_{AB}^{⊗2}\right)<0$. This approach elegantly improves the entanglement detection ability of the witnesses *W* and *V*.

**Figure 1:**
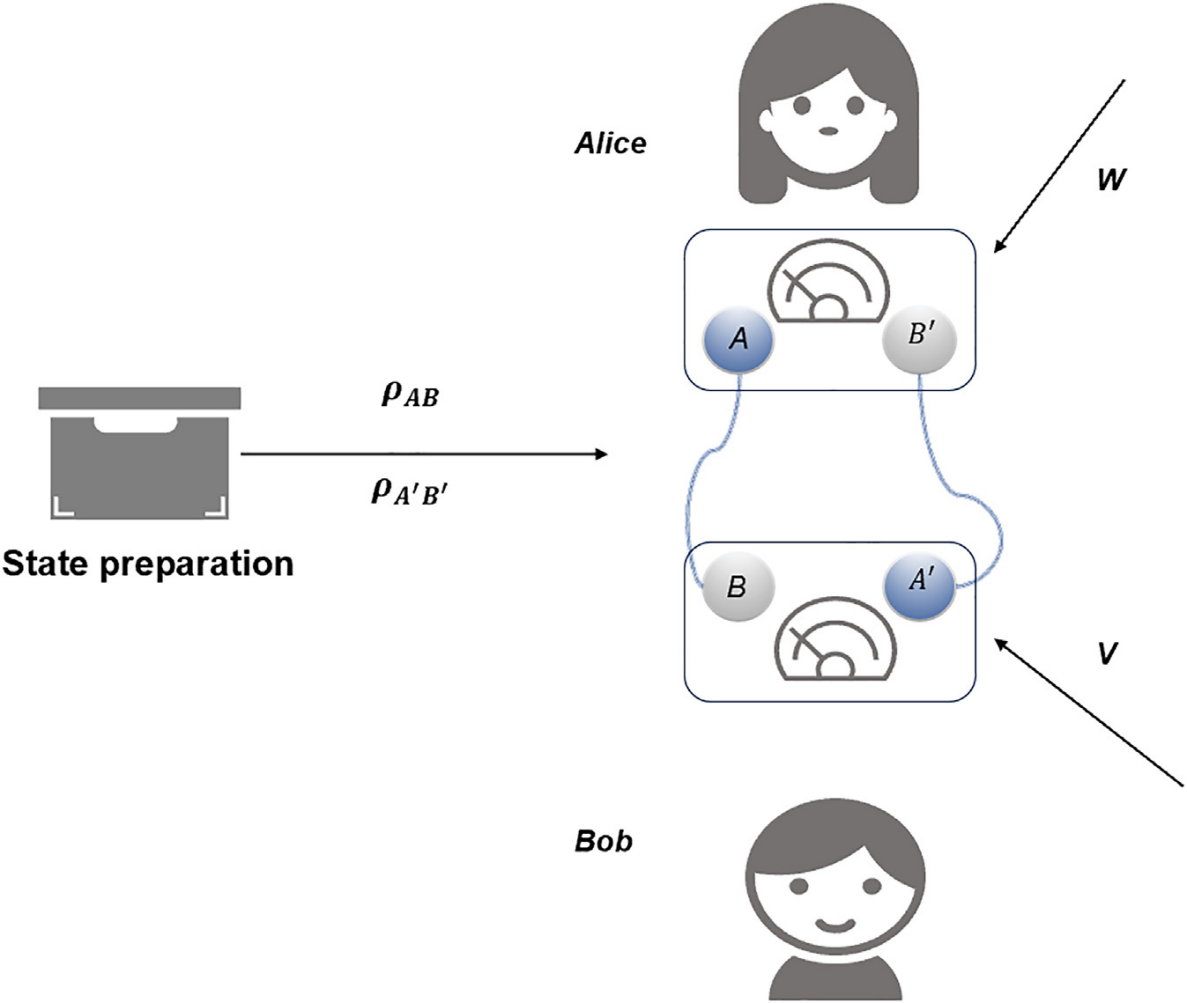
The expected value of $W_{AB^{\prime }}⊗V_{BA^{\prime }}$ is obtained by measuring W on the subsystems *A* and $B^{\prime }$, and *V* on the subsystems *B* and $A^{\prime }$

Different from the strategy given earlier, we consider further another nonlinear entanglement detection strategy. Consider two copies of a bipartite state *ρ*, $\rho_{AB}$ and $\rho_{A^{\prime }B^{\prime }}$, and two bipartite witness *W* and *V*. Bob measures the witness *V* on systems $BA^{\prime }$, and Alice measures the witness *W* on systems $AB^{\prime }$. The corresponding witness on the whole system is given by $W=W_{AB^{\prime }}⊗V_{BA^{\prime }}$. Then, the expectation value of W is given by

$${\langle W\rangle }_{\rho^{⊗2}}=\text{Tr}\left(W\left(\rho_{AB}⊗\rho_{A^{\prime }B^{\prime }}\right)\right)\text{,}$$

which is nonnegative if $\rho_{AB}$ is separable. The strategy is illustrated in Figure 1.

We have the following conclusion.

#### Observation 1

There exists an entangled state *ρ* and witnesses W and V such that Tr(Wρ)≥0 and Tr(Vρ)≥0, but $Tr\left(\left(W_{AB^{\prime }}⊗V_{BA^{\prime }}\right)\rho^{⊗2}\right)<0$.

Example 1. To illustrate the Observation 1, we consider the following 2-qubit witnesses based on the Bell state $|\varphi^{+}\rangle =\frac{1}{\sqrt{2}}\left(|10\rangle +|01\rangle \right)$ and the standard Pauli operators *X* and *Z*,

(Equation 1) $$\begin{array}{l} W=1-X⊗X+Z⊗Z\text{,}\\ V=2|\varphi^{+}\rangle {\langle \varphi^{+}|}^{\tau_{2}}\text{,} \end{array}$$

where $\tau_{2}$ stands for the partial transpose with respect to the second subsystem.

We first prove that *W* and *V* are qualified entanglement witnesses. *V* is an entanglement witness since $|\varphi^{+}\rangle$ is entangled.^52^ Assume that there exists a separable state *ρ* such that Tr(Wρ)<0. This means that $2\left(\rho_{11}+\rho_{44}\right)<\rho_{14}+\rho_{14}^{*}+\rho_{23}+\rho_{23}^{*}=2\left(Re\left(\rho_{14}\right)+Re\left(\rho_{23}\right)\right)$, namely, $\left|\rho_{14}\right|+\left|\rho_{23}\right|\geq Re\left(\rho_{14}\right)+Re\left(\rho_{23}\right)>\rho_{11}+\rho_{44}\geq 2\sqrt{\rho_{11}\rho_{44}}$. Therefore, ${\left|\rho_{23}\right|}^{2}>\rho_{11}\rho_{44}$. According to the PPT criterion,^53^ *ρ* is entangled, which contradicts to the assumption. Hence, Tr(Wρ)>0 for all separable states *ρ*. Moreover, as *W* has a negative eigenvalue of −1, *W* is a well-defined entanglement witness by the definition.^54^

To illustrate the Observation 1, we consider the Bell state $|\psi^{+}\rangle =\frac{1}{\sqrt{2}}\left(|00\rangle +|11\rangle \right)$, which gives important applications such as quantum teleportation and quantum superdense coding.^55^^,^^56^^,^^57^^,^^58^ By calculation, we have ${\langle W\rangle }_{\rho }={\langle V\rangle }_{\rho }=1$ for the state $\rho =|\psi^{+}\rangle \langle \psi^{+}|$. That is to say, the entanglement of the state *ρ* cannot be detected by either *W* or *V*. However, we have $\text{Tr}\left(\left(W_{AB^{\prime }}⊗V_{BA^{\prime }}\right)\rho^{⊗2}\right)=-\frac{1}{2}$, see the detailed calculation in Appendix A. Thus, the entanglement of *ρ* is detected by *W* and *V* together.

Next, we consider the detection of entanglement of states with non-vanishing imaginarity^59^ by using real entanglement witnesses.

Example 2. Let us consider the following quantum entangled state,

$$\sigma =\frac{1}{2}\left(\begin{array}{cccc} 0 & 0 & 0 & 0\\ 0 & 1 & i & 0\\ 0 & -i & 1 & 0\\ 0 & 0 & 0 & 0 \end{array}\right)\text{,}$$

where $i=\sqrt{-1}$. Here, due to hermitian properties, any real-valued linear witness cannot detect the entanglement of σ. In fact, it can be verified that for any real-valued entanglement witness $W_{r}$, $\text{Tr}\left(W_{r}\sigma \right)\geq 0$. Let us still use the real-valued witnesses W and V given in Example 1. By calculation, we have Tr(Wσ)=0 and Tr(Vσ)=1. Using the usual detection,^47^ one obtains $\text{Tr}\left(W_{AA^{\prime }}⊗V_{BB^{\prime }}\sigma^{⊗2}\right)=\frac{1}{2}$. However, our strategy shows that $\text{Tr}\left(W_{AB^{\prime }}⊗V_{BA^{\prime }}\sigma^{⊗2}\right)=-\frac{1}{2}$, which detects the entanglement of σ, see the detailed calculations in Appendix B.

### The case of multi-copies

Example 2 shows that our strategy is complementary to the one given previously.^47^ Moreover, when the entanglement cannot be detected with two copies of a state, we may use multi-copies.

#### Observation 2

There exists an entangled state $\rho_{ent}$ and witnesses $W_{i}$*,* i=1,2,3, such that $Tr\left(W_{i}\rho_{ent}\right)\geq 0$ for i=1,2,3 and $Tr\left(W_{i,A_{1}B_{2}}⊗W_{j,B_{1}A_{2}}\rho_{ent}^{⊗2}\right)\geq 0$ for i,j=1,2,3, but

$$Tr\left(W_{i,A_{1}B_{2}}⊗W_{j,A_{2}B_{3}}⊗W_{k,B_{1}A_{3}}\rho_{ent}^{⊗3}\right)<0$$

for some i,j,k=1,2,3.

Example 3. The Werner state,^60^

$$\rho_{w}=\frac{w1}{4}+\left(1-w\right)|\psi^{+}\rangle \langle \psi^{+}|,0\leq w\leq 1\text{,}$$

appears to be separable, entangled, and nonlocal (it violates a Bell inequality) at different parameter regions of *w*. Consider the following witnesses,

$$\begin{array}{l} W_{1}=1+X⊗X-Y⊗Y\text{,}\\ W_{2}=2|\psi^{-}\rangle {\langle \psi^{-}|}^{\tau_{2}}\text{,}\\ W_{3}=2|\psi^{+}\rangle {\langle \psi^{+}|}^{\tau_{2}}\text{,} \end{array}$$

where $|\psi^{-}\rangle =\frac{1}{\sqrt{2}}\left(|00\rangle -|11\rangle \right)$. We first show that $W_{1}$ is an entanglement witness. It can be verified that $W_{1}$ has a negative eigenvalue of −1. If $\text{Tr}\left(W_{1}\rho \right)<0$ for a separable state *ρ*, then $\rho_{11}+\rho_{22}+\rho_{33}+\rho_{44}+2\left(\rho_{14}+\rho_{14}^{*}\right)=1+4Re\left(\rho_{14}\right)<0$. Since

$$\frac{1}{4}{\left(\rho_{11}+\rho_{44}\right)}^{2}\geq \rho_{11}\rho_{44}\geq {\left|\rho_{14}\right|}^{2}\geq Re{\left(\rho_{14}\right)}^{2}>\frac{1}{16}\text{,}$$

we have $\rho_{11}+\rho_{44}>\frac{1}{2}$, and then $2\sqrt{\rho_{22}\rho_{33}}\leq \rho_{22}+\rho_{33}<1/2$. This implies that $\rho_{22}\rho_{33}<\frac{1}{16}<{\left|\rho_{14}\right|}^{2}$, and thus *ρ* is entangled based on the PPT criterion. Hence, $\text{Tr}\left(W_{1}\rho \right)\geq 0$ for all separable states *ρ*.

**Figure 2:**
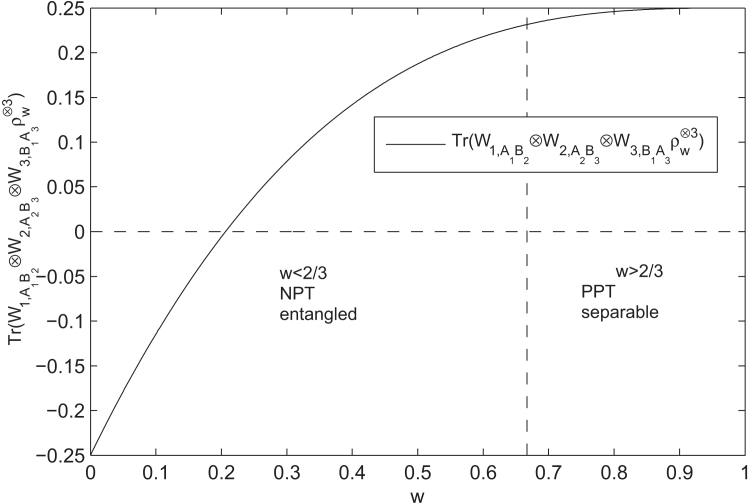
$\text{Tr}\left(W_{1,A_{1}B_{2}}⊗W_{2,A_{2}B_{3}}⊗W_{3,B_{1}A_{3}}\rho_{w}^{⊗3}\right)<0$ for w<0.206

According to the PPT criterion, $\rho_{w}$ is entangled for $w<\frac{2}{3}$. The linear detection fails to detect the entanglement of $\rho_{w}$ as $\text{Tr}\left(W_{i}\rho_{w}\right)\geq 0$, i=1,2,3. Moreover, the usual detection gives $\text{Tr}\left(W_{i,A_{1}A_{2}}⊗W_{j,B_{1}B_{2}}\rho_{w}^{⊗2}\right)\geq 0$ too. Our two-copy detection does not work either, $\text{Tr}\left(W_{i,A_{1}B_{2}}⊗W_{j,B_{1}A_{2}}\rho_{w}^{⊗2}\right)\geq 0$ for i,j=1,2,3. Note that even with three copies, the usual ordering fails to detect the entanglement of $\rho_{w}$. Nevertheless, if we use three copies of $\rho_{w}^{⊗3}$ based on our method, we obtain

$$\text{Tr}\left(W_{1,A_{1}B_{2}}⊗W_{2,A_{2}B_{3}}⊗W_{3,B_{1}A_{3}}\rho_{w}^{⊗3}\right)<0$$

for 0≤w<0.206. For instance, we have

$$\text{Tr}\left(W_{1,A_{1}B_{2}}⊗W_{2,A_{2}B_{3}}⊗W_{3,B_{1}A_{3}}{|\psi^{+}\rangle }^{⊗3}\right)=-0.25<0$$

for w=0, see Figure 2 and the details in Appendix C.

**Figure 3:**
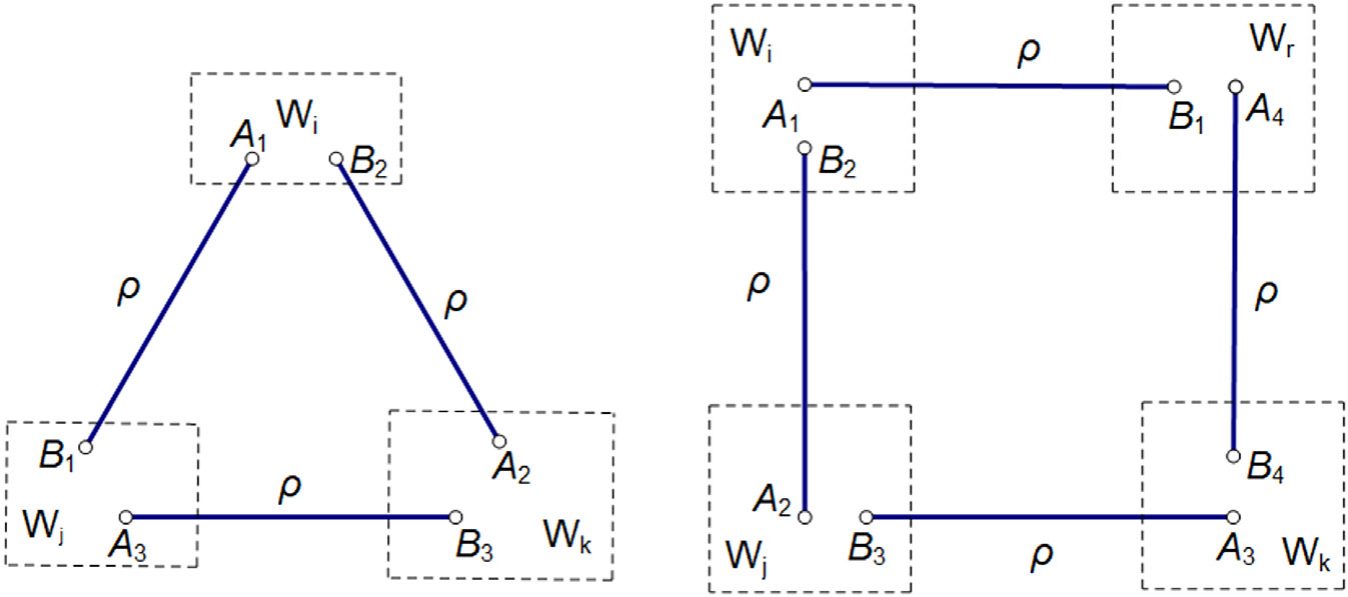
The left (right) figure shows strategy of measuring witnesses on three (four) copies of state *ρ*

Hence, our strategy applies to nonlinear detection of quantum entanglement involving general multi-copies of the quantum state *ρ*, see Figure 3 for the strategy with three and four copies by using the same or more new witnesses.

### With positive semidefinite operators

Moreover, in adopting more copies of a quantum state, although our strategy offers more new witnesses to be utilized, we may also use general observables instead of witnesses. Namely, one may replace some $W_{i}$ in $W=W_{1}⊗W_{2}⊗\ldots ⊗W_{n}$ with some positive semidefinite operators (not necessary entanglement witness). The expectation value will remain nonnegative for separable states. There exist entangled states $\rho_{ent}$ and witness *W* such that the entanglement of $\rho_{ent}$ cannot be detected *W*, i.e., $\text{Tr}\left(W\rho_{ent}\right)\geq 0$. Rico et al.^47^ showed that witnesses and positive semidefinite operator can detect entanglement in some states by using the usual ordering $A_{1}A_{2}|B_{1}B_{2}$. Based on the inspiration from Observation 1, a natural idea is the combination of witness and certain positive semidefinite operator *P* to detect the entanglement by using our strategy $A_{1}B_{2}|B_{1}A_{2}$.

#### Observation 3

For some entangled states ρ and witnesses W such that Tr(Wρ)≥0, there exists positive semidefinite operators *P* such that $Tr\left(\left(P_{AB^{\prime }}⊗W_{BA^{\prime }}\right)\rho^{⊗2}\right)<0$.

*Example 4*. Let us consider another Werner state,

$$\rho_{a}=a|\psi^{-}\rangle \langle \psi^{-}|+\frac{1-a}{4}1\text{,}$$

where 0≤a≤1. The state $\rho_{a}$ is entangled when $a>\frac{1}{3}$ according to the PPT criterion. We use the witness operator $W_{3}=2|\psi^{+}\rangle {\langle \psi^{+}|}^{\tau_{2}}$ and the positive semidefinite operator

$$P=\left(\begin{array}{cccc} 1 & 0 & 0 & -1\\ 0 & 2 & -2 & 0\\ 0 & -2 & 2 & 0\\ -1 & 0 & 0 & 1 \end{array}\right)\text{.}$$

Since $\text{Tr}\left(W_{3}\rho_{a}\right)=\frac{1+a}{2}\geq 0$, $W_{3}$ fails to detect the entanglement of $\rho_{a}$. However, taking $W=P⊗W_{3}$, we obtain $\text{Tr}\left(W\rho_{a}^{⊗2}\right)<0$ for $a>\sqrt{\frac{3}{5}}$, see the detailed calculations in Appendix D. In fact, we can improve the result by replacing *P* with

$$P_{b}=\frac{1}{4b}\left(\begin{array}{cccc} 1 & 0 & 0 & -1\\ 0 & 2b & -2b & 0\\ 0 & -2b & 2b & 0\\ -1 & 0 & 0 & 1 \end{array}\right)\text{.}$$

It is obvious that $P_{b}$ is a positive semidefinite operator for b≥1. Let $W_{b}=P_{b}⊗W_{3}$. We have

$$\text{Tr}\left(W_{b}\rho_{a}^{⊗2}\right)=\frac{\left[\left(1-6b\right)a^{2}+2b+1\right]}{16b}\text{.}$$

**Figure 4:**
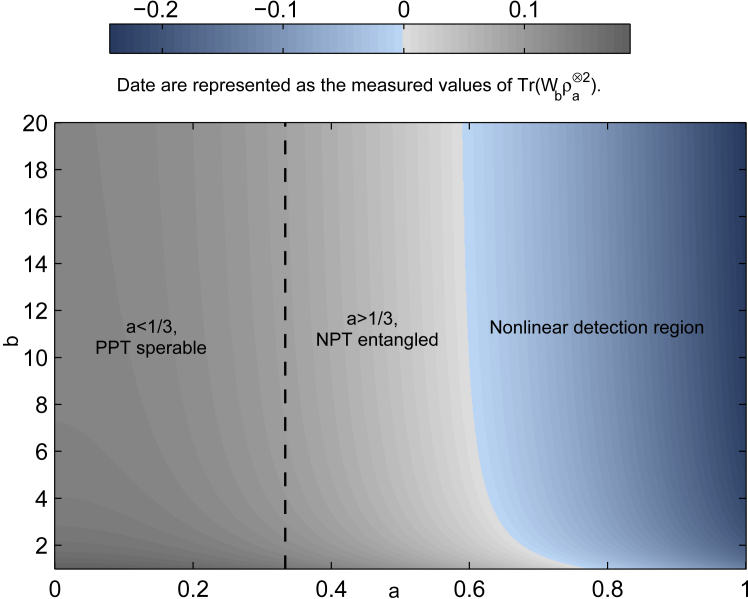
$\text{Tr}\left(W_{b}\rho_{a}^{⊗2}\right)$ versus parameters *a* and *b*

$\text{Tr}\left(W_{b}\rho_{a}^{⊗2}\right)<0$ gives rise to $a>\sqrt{\frac{2b+1}{6b-1}}$, which approaches $\sqrt{\frac{1}{3}}$ when *b* goes to +∞. Namely, the entanglement of the state is detected for $a>\sqrt{\frac{1}{3}}$, see Figure 4.

### Applied to multipartite entanglement

Furthermore, besides bipartite states, our nonlinear entanglement detection strategy may also be applied to multi-copies of multipartite quantum states with a number of witness operators, as long as the dimension of the witness operators matches the dimension of the quantum states.

#### Observation 4

There exists some tripartite entangled state $\rho_{ent}$ and bipartite witnesses $W_{i}\;\left(i=1,2,3\right)$ such that $\text{Tr}\left(W_{i}⊗W_{j}⊗W_{k}\rho_{ent}^{⊗2}\right)\geq 0$ (i,j,k=1,2,3), but

$$Tr\left(W_{i,AB^{\prime }}⊗W_{j,BC^{\prime }}⊗W_{k,CA^{\prime }}\rho_{ent}^{⊗2}\right)<0\text{,}$$

with some i,j,k∈{1,2,3}.

Observation 4 may suggest a new perspective for detecting three-qubit entangled states: measure two-qubit witnesses on two copies of the three-qubit state, with dimensions matching each other. By using the witnesses $W_{3}=|\psi^{+}\rangle {\langle \psi^{+}|}^{\tau_{2}}$ and $W_{4}=1-Z⊗Z-X⊗X$,^47^ one cannot successfully detect the entanglement of the Greenberger-Horne-Zeilinger (GHZ) state $|GHZ\rangle =1/\sqrt{2}\left(|000\rangle +|111\rangle \right)$ with two copies, under the measurement of $W_{4,AA^{\prime }}⊗W_{4,BB^{\prime }}⊗W_{3,CC^{\prime }}$,^47^ because $\text{Tr}\left(W_{4,AA^{\prime }}⊗W_{4,BB^{\prime }}⊗W_{3,CC^{\prime }}{|GHZ\rangle }^{⊗2}\right)\geq 0$. Nevertheless, we have

$$\begin{array}{l} \text{Tr}\left(W_{4,AA^{\prime }}⊗W_{3,BB^{\prime }}⊗W_{3,CC^{\prime }}{|GHZ\rangle }^{⊗2}\right)<0\text{,}\\ \text{Tr}\left(W_{4,AB^{\prime }}⊗W_{3,BC^{\prime }}⊗W_{3,CA^{\prime }}{|GHZ\rangle }^{⊗2}\right)<0\text{.} \end{array}$$

Similar consideration applies to the W states used in quantum computation and communication tasks.^61^^,^^62^^,^^63^^,^^64^

Example 5. Consider the W state mixed with white noise,

$$\rho_{c}=\left(1-c\right)|W\rangle \langle W|+\frac{c}{8}1\text{,}$$

where $|W\rangle =1/\sqrt{3}\left(|001\rangle +|010\rangle +|100\rangle \right)$ and c∈[0,1]. We use witnesses $W_{3}=2|\psi^{+}\rangle {\langle \psi^{+}|}^{\tau_{2}}$ and $W_{4}=1-Z⊗Z-X⊗X$ aforementioned. It can be verified that

$$\text{Tr}\left(W_{i}⊗W_{j}⊗W_{k}\rho_{c}^{⊗2}\right)\geq 0,i,j,k\in \left\{3,4\right\}\text{.}$$

However, by using our nonlinear detection, we have $\text{Tr}\left(\left(W_{4,AB^{\prime }}⊗W_{3,BC^{\prime }}⊗W_{3,CA^{\prime }}\right)\rho_{c}^{⊗2}\right)<0$ for c<0.406, namely, the entanglement is detected by $W_{3}$ and $W_{4}$, see the detailed calculations in Appendix E.

Bourennane et al. proposed a witness for the W state,^65^

$$W_{W}^{\left(1\right)}=\frac{2}{3}1-|W\rangle \langle W|\text{.}$$

**Figure 5:**
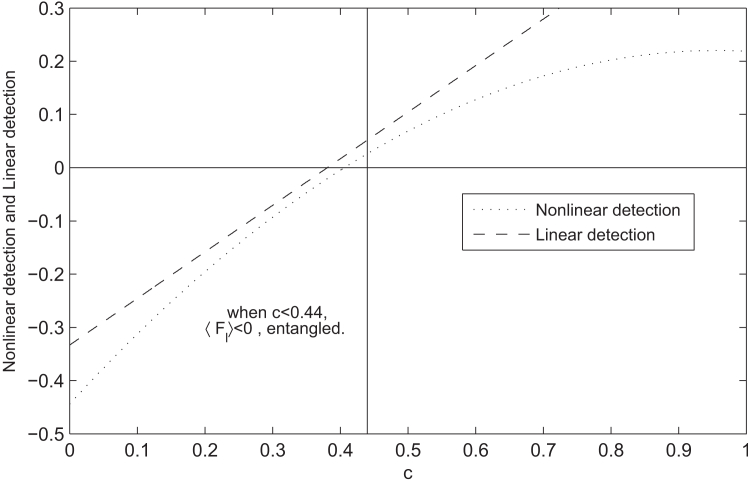
The relations between nonlinear detection and linear detection for different values of *c* It can be observed that $\text{Tr}\left(\left(W_{4,AB^{\prime }}⊗W_{3,BC^{\prime }}⊗W_{3,CA^{\prime }}\right)\rho_{c}^{⊗2}\right)<0$ for c<0.406 and $\text{Tr}\left(W_{W}^{\left(1\right)}\rho_{c}\right)<0$ for c<0.38.

It is shown that $\rho_{c}$ exhibits tripartite entanglement for c<0.38, see Figure 5. In other words, our nonlinear detection method can not only detect tripartite entanglement by using bipartite witnesses but also improve the detection ability of tripartite witness. Note that Zhao et al.^66^ introduced a tripartite entanglement criterion based on some complementary local observables and proved that $\rho_{c}$ is entangled for c<0.44.

### Applied to entanglement concentration

Our nonlinear entanglement detection strategy can also be applied to entanglement concentration. This scenario can be applied to the following situation: the two copies of |φ〉 are shared by Alice and Bob in a way that Alice has $AB^{\prime }$ and Bob has $BA^{\prime }$. Bob’s measurement concentrates the entanglement of the partition AB and $A^{\prime }B^{\prime }$ into the Alice’s subsystem $AB^{\prime }$. Furthermore, if appropriate measurements are taken, the final state on $AB^{\prime }$ can reach the maximally entangled state. Detailed scheme and steps are as follows.

Consider two copies ${|\varphi \rangle }_{AB}$ and ${|\varphi \rangle }_{A^{\prime }B^{\prime }}$ of a bipartite pure state |φ〉 with full Schmidt rank. Generally, |φ〉 can be expressed as

$$|\varphi \rangle =1⊗\Psi |\psi^{+}\rangle =\Psi^{T}⊗1|\psi^{+}\rangle \text{,}$$

where Ψ is a d×d full-ranked complex matrix such that $\text{Tr}\left(\Psi^{†}\Psi \right)=1$ and $|\psi^{+}\rangle =\frac{1}{\sqrt{d}}\sum_{i=0}^{d-1}|ii\rangle$ is a Bell state. Performing a measurement |m〉〈m| on the subsystems $BA^{\prime }$ with $|m\rangle =1⊗{\left(\Psi^{*}\right)}^{-1}|\psi^{+}\rangle$, i.e., a measurement $1_{AB^{\prime }}⊗|m\rangle {\langle m|}_{BA^{\prime }}$ on ${|\psi \rangle }^{⊗2}$, we obtain the final state |φ〉 of $AB^{\prime }$ with some probability,

$$\frac{1}{p_{m}}{\text{Tr}}_{BA^{\prime }}\left(1_{AB^{\prime }}⊗|m\rangle {\langle m|}_{BA^{\prime }}|\varphi \rangle {\langle \varphi |}^{⊗2}\right)=\frac{1}{d^{2}p_{m}}|\varphi \rangle \langle \varphi |\text{,}$$

where $p_{m}=\text{Tr}\left(1_{AB^{\prime }}⊗|m\rangle {\langle m|}_{BA^{\prime }}|\varphi \rangle {\langle \varphi |}^{⊗2}\right)$. Furthermore, if we replace the measurement acting on $BA^{\prime }$ with |M〉〈M| with $|M\rangle =1⊗{\left(\Psi^{*}\Psi^{*}\right)}^{-1}|\psi^{+}\rangle ={\left(\Psi^{†}\right)}^{-1}⊗{\left(\Psi^{*}\right)}^{-1}|\psi^{+}\rangle$, then we get the state

$$\frac{1}{p_{m}^{\prime }}{\text{Tr}}_{BA^{\prime }}\left(1_{AB^{\prime }}⊗|M\rangle {\langle M|}_{BA^{\prime }}|\varphi \rangle {\langle \varphi |}^{⊗2}\right)=\frac{1}{d^{2}p_{m}^{\prime }}|\psi^{+}\rangle \langle \psi^{+}|\text{,}$$

where $p_{m}^{\prime }=\text{Tr}\left(1_{AB^{\prime }}⊗|M\rangle {\langle M|}_{BA^{\prime }}|\varphi \rangle {\langle \varphi |}^{⊗2}\right)$, see the detailed derivation in Appendix F. Therefore, our strategy provides an effective approach of entanglement concentration.

## Discussion

In complementary to the one given earlier,^47^ we have presented a different nonlinear entanglement detection strategy, which detects entanglement that the linear entanglement detection strategy fails. Meanwhile, we have generalized the strategy by adopting multi-copies and shown that when the detection for two copies fails, the detection for three or more copies can be successful, providing a new perspective for entanglement detection. Moreover, we have shown that there exist pairs of states and witnesses such that linear detection strategy fails, the nonlinear detection strategy given by combining the witness with a quantum mechanical observable succeeds, and the entanglement detection ability can be improved by adjusting the observables. Furthermore, our strategy can also be applied to entanglement concentrations. Such nonlinear entanglement detection strategy is a new avenue. Our results may also highlight investigations on detections of other kind of quantum correlations.

### Limitations of the study

The paper presents a nonlinear entanglement strategy that detects entanglement that the linear witness detection fails. Although we have considered the nonlinear detection of Bell state, Werner state, imaginary state, GHZ state, and W state in this work, with some of them mixed with the white noise, the main limitation of the nonlinear method is that there are no more general results on this issue at present.

## Resource availability

### Lead contact

Further information and other requests should be directed to and fulfilled by the lead contact, Tinggui Zhang (tinggui333@163.com).

### Materials availability

This work did not generate any new materials.

### Data and code availability

• All data reported in this paper will be shared by the lead contact upon request.
• This paper does not report original code.
• Any additional information required to reanalyze the data reported in this paper is available from the lead contact upon request.

## Acknowledgments

This work was supported by the 10.13039/501100001809National Natural Science Foundation of China under grant nos. 12204137, 12075159, and 12171044 and the specific research fund of the Innovation Platform for Academicians of Hainan Province.

## Author contributions

Y.W., T.Z., and X.H.: conceptualization, methodology, validation, investigation, and writing – original draft; T.Z. and S.-M.F.: writing – review and editing and supervision.

## Declaration of interests

The authors declare no competing interests.

## STAR★Methods

### Key resources table

**Table:** 

REAGENT or RESOURCE	SOURCE	IDENTIFIER
Linear entanglement witnesses detection	Gühne et al.^15^	RRID: AB_2313773
Nonlinear entanglement witnesses detection	Gühne et al.^15^	RRID: AB_2313773
partial transpose criterion	Peres^53^	RRID: AB_2313773
Entanglement detection with trace polynomials	Rico et al.^47^	RRID: AB_2313773
Witness based nonlinear detection of quantum entanglement	This paper	RRID: AB_2313773

### Method details

#### The details of example 1

Utilizing our strategy, we write down each term of $\rho_{AB}^{⊗2}$ after measuring the operators $W_{AB^{\prime }}$ and $V_{BA^{\prime }}$. Without causing confusion, we abbreviate ${|0\rangle }_{A}{|0\rangle }_{B}{|0\rangle }_{A}^{\prime }{|0\rangle }_{B}^{\prime }$ as |0000〉, and similarly for other terms.

$$\begin{array}{cc} |0000\rangle \langle 0000|\overset{W_{AB^{\prime }}}{\underset{V_{BA^{\prime }}}{\to }}2|0110\rangle \langle 0000|-|1111\rangle \langle 0000|\text{,} & |0000\rangle \langle 0011|\overset{W_{AB^{\prime }}}{\underset{V_{BA^{\prime }}}{\to }}2|0110\rangle \langle 0011|-|1111\rangle \langle 0011|\text{,}\\ |0000\rangle \langle 1100|\overset{W_{AB^{\prime }}}{\underset{V_{BA^{\prime }}}{\to }}2|0110\rangle \langle 1100|-|1111\rangle \langle 1100|\text{,} & ★|0000\rangle \langle 1111|\overset{W_{AB^{\prime }}}{\underset{V_{BA^{\prime }}}{\to }}2|0110\rangle \langle 1111|-|1111\rangle \langle 1111|\text{,}\\ |0011\rangle \langle 0000|\overset{W_{AB^{\prime }}}{\underset{V_{BA^{\prime }}}{\to }}-|1010\rangle \langle 0000|\text{,} & |0011\rangle \langle 0011|\overset{W_{AB^{\prime }}}{\underset{V_{BA^{\prime }}}{\to }}-|1010\rangle \langle 0011|\text{,}\\ |0011\rangle \langle 1100|\overset{W_{AB^{\prime }}}{\underset{V_{BA^{\prime }}}{\to }}-|1010\rangle \langle 1100|\text{,} & |0011\rangle \langle 1111|\overset{W_{AB^{\prime }}}{\underset{V_{BA^{\prime }}}{\to }}-|1010\rangle \langle 1111|\text{,}\\ |1100\rangle \langle 0000|\overset{W_{AB^{\prime }}}{\underset{V_{BA^{\prime }}}{\to }}-|0101\rangle \langle 0000|\text{,} & |1100\rangle \langle 0011|\overset{W_{AB^{\prime }}}{\underset{V_{BA^{\prime }}}{\to }}-|0101\rangle \langle 0011|\text{,}\\ |1100\rangle \langle 1100|\overset{W_{AB^{\prime }}}{\underset{V_{BA^{\prime }}}{\to }}-|0101\rangle \langle 1100|\text{,} & |1100\rangle \langle 1111|\overset{W_{AB^{\prime }}}{\underset{V_{BA^{\prime }}}{\to }}-|0101\rangle \langle 1111|\text{,}\\ ★|1111\rangle \langle 0000|\overset{W_{AB^{\prime }}}{\underset{V_{BA^{\prime }}}{\to }}2|1001\rangle \langle 0000|-|0000\rangle \langle 0000|\text{,} & |1111\rangle \langle 0011|\overset{W_{AB^{\prime }}}{\underset{V_{BA^{\prime }}}{\to }}2|1001\rangle \langle 0011|-|0000\rangle \langle 0011|\text{,}\\ |1111\rangle \langle 1100|\overset{W_{AB^{\prime }}}{\underset{V_{BA^{\prime }}}{\to }}2|1001\rangle \langle 1100|-|0000\rangle \langle 1100|\text{,} & |1111\rangle \langle 1111|\overset{W_{AB^{\prime }}}{\underset{V_{BA^{\prime }}}{\to }}2|1001\rangle \langle 1111|-|0000\rangle \langle 1111|\text{,} \end{array}$$

There are only two elements appearing on the diagonal (marked with a pentagram). The corresponding coefficients are all $-\frac{1}{4}$. Thus we have $S_{1}=\text{Tr}\left(\left(W_{AB^{\prime }}⊗V_{BA^{\prime }}\right)\rho_{AB}^{⊗2}\right)=-\frac{1}{4}+\left(-\frac{1}{4}\right)=-\frac{1}{2}$, measurement results of two copies of Bell state using witnesses *W* and *V* based on our nonlinear strategy.

#### The details of example 2

Using the usual ordering strategy, one obtains

$$\begin{array}{cc} |0101\rangle \langle 0101|\overset{W_{AA^{\prime }}}{\underset{V_{BB^{\prime }}}{\to }}2|0000\rangle \langle 0101|-|1010\rangle \langle 0101|\text{,} & |0110\rangle \langle 0101|\overset{W_{AA^{\prime }}}{\underset{V_{BB^{\prime }}}{\to }}-|1100\rangle \langle 0101|\text{,}\\ |0101\rangle \langle 0110|\overset{W_{AA^{\prime }}}{\underset{V_{BB^{\prime }}}{\to }}2|0000\rangle \langle 0110|-|1010\rangle \langle 0110|\text{,} & |0110\rangle \langle 0110|\overset{W_{AA^{\prime }}}{\underset{V_{BB^{\prime }}}{\to }}-|1100\rangle \langle 0110|\text{,}\\ |0101\rangle \langle 1001|\overset{W_{AA^{\prime }}}{\underset{V_{BB^{\prime }}}{\to }}2|0000\rangle \langle 1001|-|1010\rangle \langle 1001|\text{,} & |0110\rangle \langle 1001|\overset{W_{AA^{\prime }}}{\underset{V_{BB^{\prime }}}{\to }}-|1100\rangle \langle 1001|\text{,}\\ ★|0101\rangle \langle 1010|\overset{W_{AA^{\prime }}}{\underset{V_{BB^{\prime }}}{\to }}2|0000\rangle \langle 1010|-|1010\rangle \langle 1010|\text{,} & |0110\rangle \langle 1010|\overset{W_{AA^{\prime }}}{\underset{V_{BB^{\prime }}}{\to }}-|1100\rangle \langle 1010|\text{,}\\ ★|1010\rangle \langle 0101|\overset{W_{AA^{\prime }}}{\underset{V_{BB^{\prime }}}{\to }}2|1111\rangle \langle 0101|-|0101\rangle \langle 0101|\text{,} & |1001\rangle \langle 0101|\overset{W_{AA^{\prime }}}{\underset{V_{BB^{\prime }}}{\to }}-|0011\rangle \langle 0101|\text{,}\\ |1010\rangle \langle 0110|\overset{W_{AA^{\prime }}}{\underset{V_{BB^{\prime }}}{\to }}2|1111\rangle \langle 0110|-|0101\rangle \langle 0110|\text{,} & |1001\rangle \langle 0110|\overset{W_{AA^{\prime }}}{\underset{V_{BB^{\prime }}}{\to }}-|0011\rangle \langle 0110|\text{,}\\ |1010\rangle \langle 1001|\overset{W_{AA^{\prime }}}{\underset{V_{BB^{\prime }}}{\to }}2|1111\rangle \langle 1001|-|0101\rangle \langle 1001|\text{,} & |1001\rangle \langle 1001|\overset{W_{AA^{\prime }}}{\underset{V_{BB^{\prime }}}{\to }}-|0011\rangle \langle 1001|\text{,}\\ |1010\rangle \langle 1010|\overset{W_{AA^{\prime }}}{\underset{V_{BB^{\prime }}}{\to }}2|1111\rangle \langle 1010|-|0101\rangle \langle 1010|\text{,} & |1001\rangle \langle 1010|\overset{W_{AA^{\prime }}}{\underset{V_{BB^{\prime }}}{\to }}-|0011\rangle \langle 1010|\text{.} \end{array}$$

So we have $S_{2}=\text{Tr}\left(W_{AA^{\prime }}⊗V_{BB^{\prime }}\sigma^{⊗2}\right)=-{\left(\frac{i}{2}\right)}^{2}-{\left(-\frac{i}{2}\right)}^{2}=\frac{1}{4}+\frac{1}{4}=\frac{1}{2}$, measurement results of two copies of *σ* using witnesses *W* and *V* based on usual ordering strategy. However, our nonlinear strategy gives

$$\begin{array}{cc} |0110\rangle \langle 0101|\overset{W_{AB^{\prime }}}{\underset{V_{BA^{\prime }}}{\to }}2|0000\rangle \langle 0101|-|1001\rangle \langle 0101|\text{,} & |0101\rangle \langle 0101|\overset{W_{AB^{\prime }}}{\underset{V_{BA^{\prime }}}{\to }}-|1100\rangle \langle 0101|\text{,}\\ ★|0110\rangle \langle 1001|\overset{W_{AB^{\prime }}}{\underset{V_{BA^{\prime }}}{\to }}2|0000\rangle \langle 1001|-|1001\rangle \langle 1001|\text{,} & |0101\rangle \langle 0110|\overset{W_{AB^{\prime }}}{\underset{V_{BA^{\prime }}}{\to }}-|1100\rangle \langle 0110|\text{,}\\ |0110\rangle \langle 0110|\overset{W_{AB^{\prime }}}{\underset{V_{BA^{\prime }}}{\to }}2|0000\rangle \langle 0110|-|1001\rangle \langle 0110|\text{,} & |0101\rangle \langle 1001|\overset{W_{AB^{\prime }}}{\underset{V_{BA^{\prime }}}{\to }}-|1100\rangle \langle 1001|\text{,}\\ |0110\rangle \langle 1010|\overset{W_{AB^{\prime }}}{\underset{V_{BA^{\prime }}}{\to }}2|0000\rangle \langle 1010|-|1001\rangle \langle 1010|\text{,} & |0101\rangle \langle 1010|\overset{W_{AB^{\prime }}}{\underset{V_{BA^{\prime }}}{\to }}-|1100\rangle \langle 1010|\text{,}\\ |1001\rangle \langle 0101|\overset{W_{AB^{\prime }}}{\underset{V_{BA^{\prime }}}{\to }}2|1111\rangle \langle 0101|-|0110\rangle \langle 0101|\text{,} & |1010\rangle \langle 0101|\overset{W_{AB^{\prime }}}{\underset{V_{BA^{\prime }}}{\to }}-|0011\rangle \langle 0101|\text{,}\\ ★|1001\rangle \langle 0110|\overset{W_{AB^{\prime }}}{\underset{V_{BA^{\prime }}}{\to }}2|1111\rangle \langle 0110|-|0110\rangle \langle 0110|\text{,} & |1010\rangle \langle 0110|\overset{W_{AB^{\prime }}}{\underset{V_{BA^{\prime }}}{\to }}-|0011\rangle \langle 0110|\text{,}\\ |1001\rangle \langle 1001|\overset{W_{AB^{\prime }}}{\underset{V_{BA^{\prime }}}{\to }}2|1111\rangle \langle 1001|-|0110\rangle \langle 1001|\text{,} & |1010\rangle \langle 1001|\overset{W_{AB^{\prime }}}{\underset{V_{BA^{\prime }}}{\to }}-|0011\rangle \langle 1001|\text{,}\\ |1001\rangle \langle 1010|\overset{W_{AB^{\prime }}}{\underset{V_{BA^{\prime }}}{\to }}2|1111\rangle \langle 1010|-|0110\rangle \langle 1010|\text{,} & |1010\rangle \langle 1010|\overset{W_{AB^{\prime }}}{\underset{V_{BA^{\prime }}}{\to }}-|0011\rangle \langle 1010|\text{.} \end{array}$$

Therefore, $S_{3}=\text{Tr}\left(W_{AB^{\prime }}⊗V_{BA^{\prime }}\sigma^{⊗2}\right)=-\left(\frac{i}{2}.\frac{-i}{2}\right)-\left(\frac{-i}{2}.\frac{i}{2}\right)=-\frac{1}{4}-\frac{1}{4}=-\frac{1}{2}$, measurement results of two copies of *σ* using witnesses *W* and *V* based on our nonlinear strategy.

#### The details of example 3

We calculate the value of $S_{4}=\text{Tr}\left(W_{1,A_{1}B_{2}}⊗W_{2,A_{2}B_{3}}⊗W_{2,B_{1}A_{3}}\rho_{w}^{⊗3}\right)$, measurement results of three copies of Werner state using witnesses $W_{1}$, $W_{2}$ and $W_{3}$ based on our nonlinear strategy, related to Figures 2 and 3. For simplicity, we only list the terms that contribute to the trace,

$$\begin{array}{l} |000000\rangle \langle 000000|\overset{W_{1,A_{1}B_{2}}}{\underset{W_{2,A_{2}B_{3}},W_{3,B_{1}A_{3}}}{\to }}|000000\rangle \langle 000000|+2|100100\rangle \langle 000000|\text{,}\\ |000011\rangle \langle 111100|\overset{W_{1,A_{1}B_{2}}}{\underset{W_{2,A_{2}B_{3}},W_{3,B_{1}A_{3}}}{\to }}-|011000\rangle \langle 111100|-2|111100\rangle \langle 111100|\text{,}\\ |111100\rangle \langle 000011|\overset{W_{1,A_{1}B_{2}}}{\underset{W_{2,A_{2}B_{3}},W_{3,B_{1}A_{3}}}{\to }}-|100111\rangle \langle 000011|-2|000011\rangle \langle 000011|\text{,}\\ |111111\rangle \langle 111111|\overset{W_{1,A_{1}B_{2}}}{\underset{W_{2,A_{2}B_{3}},W_{3,B_{1}A_{3}}}{\to }}|111111\rangle \langle 111111|+2|011011\rangle \langle 111111|\text{.}\\ |001001\rangle \langle 001001|\overset{W_{1,A_{1}B_{2}}}{\underset{W_{2,A_{2}B_{3}},W_{3,B_{1}A_{3}}}{\to }}|001001\rangle \langle 001001|+2|101101\rangle \langle 001001|\text{,}\\ |010010\rangle \langle 010010|\overset{W_{1,A_{1}B_{2}}}{\underset{W_{2,A_{2}B_{3}},W_{3,B_{1}A_{3}}}{\to }}|010010\rangle \langle 010010|+2|110110\rangle \langle 010010|\text{,}\\ |011011\rangle \langle 011011|\overset{W_{1,A_{1}B_{2}}}{\underset{W_{2,A_{2}B_{3}},W_{3,B_{1}A_{3}}}{\to }}|011011\rangle \langle 011011|+2|111111\rangle \langle 011011|\text{,}\\ |100100\rangle \langle 100100|\overset{W_{1,A_{1}B_{2}}}{\underset{W_{2,A_{2}B_{3}},W_{3,B_{1}A_{3}}}{\to }}|100100\rangle \langle 100100|+2|000000\rangle \langle 100100|\text{,}\\ |101101\rangle \langle 101101|\overset{W_{1,A_{1}B_{2}}}{\underset{W_{2,A_{2}B_{3}},W_{3,B_{1}A_{3}}}{\to }}|101101\rangle \langle 101101|+2|001001\rangle \langle 101101|\text{,}\\ |110110\rangle \langle 110110|\overset{W_{1,A_{1}B_{2}}}{\underset{W_{2,A_{2}B_{3}},W_{3,B_{1}A_{3}}}{\to }}|110110\rangle \langle 110110|+2|010010\rangle \langle 110110|\text{,}\\ |000100\rangle \langle 000100|\overset{W_{1,A_{1}B_{2}}}{\underset{W_{2,A_{2}B_{3}},W_{3,B_{1}A_{3}}}{\to }}|000100\rangle \langle 000100|,|001101\rangle \langle 001101|\overset{W_{1,A_{1}B_{2}}}{\underset{W_{2,A_{2}B_{3}},W_{3,B_{1}A_{3}}}{\to }}|001101\rangle \langle 001101|\text{,} \end{array}$$

(Equation 2) $$\begin{array}{cc} |010110\rangle \langle 010110|\overset{W_{1,A_{1}B_{2}}}{\underset{W_{2,A_{2}B_{3}},W_{3,B_{1}A_{3}}}{\to }}|010110\rangle \langle 010110|\text{,} & |011111\rangle \langle 011111|\overset{W_{1,A_{1}B_{2}}}{\underset{W_{2,A_{2}B_{3}},W_{3,B_{1}A_{3}}}{\to }}|011111\rangle \langle 011111|\text{,}\\ |111011\rangle \langle 111011|\overset{W_{1,A_{1}B_{2}}}{\underset{W_{2,A_{2}B_{3}},W_{3,B_{1}A_{3}}}{\to }}|111011\rangle \langle 111011|\text{,} & |110010\rangle \langle 110010|\overset{W_{1,A_{1}B_{2}}}{\underset{W_{2,A_{2}B_{3}},W_{3,B_{1}A_{3}}}{\to }}|110010\rangle \langle 110010|\text{,}\\ |101001\rangle \langle 101001|\overset{W_{1,A_{1}B_{2}}}{\underset{W_{2,A_{2}B_{3}},W_{3,B_{1}A_{3}}}{\to }}|101001\rangle \langle 101001|\text{,} & |100000\rangle \langle 100000|\overset{W_{1,A_{1}B_{2}}}{\underset{W_{2,A_{2}B_{3}},W_{3,B_{1}A_{3}}}{\to }}|100000\rangle \langle 100000|\text{,} \end{array}$$

Therefore,

$$S_{4}=\text{Tr}\left(W_{1,A_{1}B_{2}}⊗W_{2,A_{2}B_{3}}⊗W_{3,B_{1}A_{3}}\rho_{w}^{⊗3}\right)=2{\left(\frac{2-w}{4}\right)}^{3}-4{\left(\frac{1-w}{2}\right)}^{3}+6\frac{w{\left(2-w\right)}^{2}}{64}+6\frac{w^{2}\left(2-w\right)}{64}+2\frac{w^{3}}{64}\text{,}$$

from which it is derived that $\text{Tr}\left(W_{1,A_{1}B_{2}}⊗W_{2,A_{2}B_{3}}⊗W_{3,B_{1}A_{3}}\rho_{w}^{⊗3}\right)<0$ when w<0.206. In particular, when w=0 we have $S_{5}=\text{Tr}\left(W_{1,A_{1}B_{2}}⊗W_{2,A_{2}B_{3}}⊗W_{3,B_{1}A_{3}}{|\psi^{+}\rangle }^{⊗3}\right)=-0.25<0$, measurement results of three copies of Werner state based on our nonlinear strategy for w=0, related to Figure 2.

#### The details of example 4

Concerning the calculation of $S_{6}=\text{Tr}\left(W\rho_{a}^{⊗2}\right)$, measurement results of two copies of Werner state $\rho_{a}$ using witness operator $W_{3}$ with positive semidenite operator *P* based on our nonlinear strategy,

$$\begin{array}{cc} ★|0000\rangle \langle 0000|\overset{P_{AB^{\prime }}}{\underset{W_{BA^{\prime }}}{\to }}|0000\rangle \langle 0000|-|1001\rangle \langle 0000|\text{,} & |0100\rangle \langle 0100|\overset{P_{AB^{\prime }}}{\underset{W_{BA^{\prime }}}{\to }}|0010\rangle \langle 0100|-|1011\rangle \langle 0100|\text{,}\\ |0000\rangle \langle 0011|\overset{P_{AB^{\prime }}}{\underset{W_{BA^{\prime }}}{\to }}|0000\rangle \langle 0011|-|1001\rangle \langle 0011|\text{,} & |0100\rangle \langle 0111|\overset{P_{AB^{\prime }}}{\underset{W_{BA^{\prime }}}{\to }}|0010\rangle \langle 0111|-|1011\rangle \langle 0111|\text{,}\\ |0000\rangle \langle 1100|\overset{P_{AB^{\prime }}}{\underset{W_{BA^{\prime }}}{\to }}|0000\rangle \langle 1100|-|1001\rangle \langle 1100|\text{,} & |0101\rangle \langle 0101|\overset{P_{AB^{\prime }}}{\underset{W_{BA^{\prime }}}{\to }}2|0011\rangle \langle 0101|-2|1010\rangle \langle 0101|\text{,}\\ |0000\rangle \langle 1111|\overset{P_{AB^{\prime }}}{\underset{W_{BA^{\prime }}}{\to }}|0000\rangle \langle 1111|-|1001\rangle \langle 1111|\text{,} & ★|0110\rangle \langle 0110|\overset{P_{AB^{\prime }}}{\underset{W_{BA^{\prime }}}{\to }}|0110\rangle \langle 0110|-|1111\rangle \langle 0110|\text{,}\\ |0011\rangle \langle 0000|\overset{P_{AB^{\prime }}}{\underset{W_{BA^{\prime }}}{\to }}2|0101\rangle \langle 0000|-2|1100\rangle \langle 0000|\text{,} & |0111\rangle \langle 0100|\overset{P_{AB^{\prime }}}{\underset{W_{BA^{\prime }}}{\to }}2|0111\rangle \langle 0100|-2|1110\rangle \langle 0100|\text{,}\\ |0011\rangle \langle 0011|\overset{P_{AB^{\prime }}}{\underset{W_{BA^{\prime }}}{\to }}2|0101\rangle \langle 0011|-2|1100\rangle \langle 0011|\text{,} & ★|0111\rangle \langle 0111|\overset{P_{AB^{\prime }}}{\underset{W_{BA^{\prime }}}{\to }}2|0111\rangle \langle 0111|-2|1110\rangle \langle 0111|\text{,}\\ ★|0011\rangle \langle 1100|\overset{P_{AB^{\prime }}}{\underset{W_{BA^{\prime }}}{\to }}2|0101\rangle \langle 1100|-2|1100\rangle \langle 1100|\text{,} & ★|1000\rangle \langle 1000|\overset{P_{AB^{\prime }}}{\underset{W_{BA^{\prime }}}{\to }}2|1000\rangle \langle 1000|-2|0001\rangle \langle 1000|\text{,}\\ |0011\rangle \langle 1111|\overset{P_{AB^{\prime }}}{\underset{W_{BA^{\prime }}}{\to }}2|0101\rangle \langle 1111|-2|1100\rangle \langle 1111|\text{,} & |1000\rangle \langle 1011|\overset{P_{AB^{\prime }}}{\underset{W_{BA^{\prime }}}{\to }}2|1000\rangle \langle 1011|-2|0001\rangle \langle 1011|\text{,}\\ |1100\rangle \langle 0000|\overset{P_{AB^{\prime }}}{\underset{W_{BA^{\prime }}}{\to }}2|1010\rangle \langle 0000|-2|0011\rangle \langle 0000|\text{,} & ★|1001\rangle \langle 1001|\overset{P_{AB^{\prime }}}{\underset{W_{BA^{\prime }}}{\to }}|1001\rangle \langle 1001|-|0000\rangle \langle 1001|\text{,}\\ ★|1100\rangle \langle 0011|\overset{P_{AB^{\prime }}}{\underset{W_{BA^{\prime }}}{\to }}2|1010\rangle \langle 0011|-2|0011\rangle \langle 0011|\text{,} & |1010\rangle \langle 1010|\overset{P_{AB^{\prime }}}{\underset{W_{BA^{\prime }}}{\to }}2|1100\rangle \langle 1010|-2|0101\rangle \langle 1010|\text{,} \end{array}$$

(Equation 3) $$\begin{array}{cc} |1100\rangle \langle 1100|\overset{P_{AB^{\prime }}}{\underset{W_{BA^{\prime }}}{\to }}2|1010\rangle \langle 1100|-2|0011\rangle \langle 1100|\text{,} & |1011\rangle \langle 1000|\overset{P_{AB^{\prime }}}{\underset{W_{BA^{\prime }}}{\to }}|1101\rangle \langle 1000|-\langle 0100|\langle 1000|\text{,}\\ |1100\rangle \langle 1111|\overset{P_{AB^{\prime }}}{\underset{W_{BA^{\prime }}}{\to }}2|1010\rangle \langle 1111|-2|0011\rangle \langle 1111|\text{,} & |1011\rangle \langle 1011|\overset{P_{AB^{\prime }}}{\underset{W_{BA^{\prime }}}{\to }}|1101\rangle \langle 1011|-|0100\rangle \langle 1011|\text{,}\\ |1111\rangle \langle 0000|\overset{P_{AB^{\prime }}}{\underset{W_{BA^{\prime }}}{\to }}|1111\rangle \langle 0000|-|0110\rangle \langle 0000|\text{,} & |1101\rangle \langle 0001|\overset{P_{AB^{\prime }}}{\underset{W_{BA^{\prime }}}{\to }}|1011\rangle \langle 0001|-|0010\rangle \langle 0001|\text{,}\\ |1111\rangle \langle 0011|\overset{P_{AB^{\prime }}}{\underset{W_{BA^{\prime }}}{\to }}|1111\rangle \langle 0011|-|0110\rangle \langle 0011|\text{,} & |1110\rangle \langle 0010|\overset{P_{AB^{\prime }}}{\underset{W_{BA^{\prime }}}{\to }}2|1110\rangle \langle 0010|-2|0111\rangle \langle 0010|\text{,}\\ |1111\rangle \langle 1100|\overset{P_{AB^{\prime }}}{\underset{W_{BA^{\prime }}}{\to }}|1111\rangle \langle 1100|-|0110\rangle \langle 1100|\text{,} & ★|1110\rangle \langle 1110|\overset{P_{AB^{\prime }}}{\underset{W_{BA^{\prime }}}{\to }}2|1110\rangle \langle 1110|-2|0111\rangle \langle 1110|\text{,}\\ ★|1111\rangle \langle 1111|\overset{P_{AB^{\prime }}}{\underset{W_{BA^{\prime }}}{\to }}|1111\rangle \langle 1111|-|0110\rangle \langle 1111|\text{,} & |1101\rangle \langle 1101|\overset{P_{AB^{\prime }}}{\underset{W_{BA^{\prime }}}{\to }}|1011\rangle \langle 1101|-|0010\rangle \langle 1101|\text{,}\\ ★|0001\rangle \langle 0001|\overset{P_{AB^{\prime }}}{\underset{W_{BA^{\prime }}}{\to }}2|0001\rangle \langle 0001|-2|1000\rangle \langle 0001|\text{,} & |0010\rangle \langle 0010|\overset{P_{AB^{\prime }}}{\underset{W_{BA^{\prime }}}{\to }}|0100\rangle \langle 0010|-|1101\rangle \langle 0010|\text{,}\\ |0001\rangle \langle 1101|\overset{P_{AB^{\prime }}}{\underset{W_{BA^{\prime }}}{\to }}2|0001\rangle \langle 1101|-2|1000\rangle \langle 1101|\text{,} & |0010\rangle \langle 1110|\overset{P_{AB^{\prime }}}{\underset{W_{BA^{\prime }}}{\to }}|0100\rangle \langle 1110|-|1101\rangle \langle 1110|\text{,} \end{array}$$

where the elements that contribute to the trace have been marked by a pentagram. Then we have

$$S_{6}=\text{Tr}\left(W\rho_{a}^{⊗2}\right)=\frac{{\left(1+a\right)}^{2}}{8}-a^{2}+\frac{1-a^{2}}{2}+\frac{{\left(1-a\right)}^{2}}{8}=\frac{3-5a^{2}}{4}\text{,}$$

related to Figure 4.

#### The details of example 5

We first show that

$$\text{Tr}\left(W_{i}⊗W_{j}⊗W_{k}\rho_{c}^{⊗2}\right)\geq 0,i,j,k\in \left\{3,4\right\}\text{.}$$

$$\begin{array}{l} \text{Tr}\left(W_{3}⊗W_{3}⊗W_{3}\rho_{c}^{⊗2}\right)=\left(\frac{1}{3}-\frac{c}{3}\right)\left(\frac{4}{3}-\frac{c}{3}\right)+\left(\frac{1}{3}-\frac{5c}{24}\right)\left(\frac{1}{3}+\frac{c}{24}\right)+\frac{5c^{2}}{64}\geq 0\text{,}\\ \text{Tr}\left(W_{3}⊗W_{3}⊗W_{4}\rho_{c}^{⊗2}\right)=\left(\frac{1}{3}-\frac{c}{3}\right)\left(\frac{4}{3}-\frac{c}{3}\right)+c\left(\frac{1}{3}-\frac{7c}{48}\right)+\frac{c^{2}}{16}\geq 0\text{,}\\ \text{Tr}\left(W_{3}⊗W_{4}⊗W_{3}\rho_{c}^{⊗2}\right)=2\left(\frac{1}{3}-\frac{c}{3}\right)\left(\frac{4}{3}-\frac{c}{3}\right)+c\left(\frac{1}{3}-\frac{5c}{24}\right)+\frac{c^{2}}{8}\geq 0\text{,}\\ \text{Tr}\left(W_{3}⊗W_{4}⊗W_{4}\rho_{c}^{⊗2}\right)=\left(\frac{1}{3}-\frac{5c}{24}\right)\left(\frac{4}{3}-\frac{c}{3}\right)+\frac{c\left(1-c\right)}{2}+c\left(\frac{1}{3}-\frac{5c}{24}\right)+\frac{c^{2}}{4}\geq 0\text{,}\\ \text{Tr}\left(W_{4}⊗W_{3}⊗W_{3}\rho_{c}^{⊗2}\right)=\left(\frac{1}{3}-\frac{c}{3}\right)\left(\frac{4}{3}-\frac{c}{3}\right)+\frac{c}{2}\left(\frac{2}{3}-\frac{7c}{24}\right)+\frac{c^{2}}{16}\geq 0\text{,}\\ \text{Tr}\left(W_{4}⊗W_{3}⊗W_{4}\rho_{c}^{⊗2}\right)=4{\left[\left(\frac{1}{3}-\frac{c}{3}\right)+2\left(\frac{1}{3}-\frac{5c}{24}\right)\right]}^{2}+\frac{c^{2}}{4}\geq 0\text{,}\\ \text{Tr}\left(W_{4}⊗W_{4}⊗W_{3}\rho_{c}^{⊗2}\right)=\left(\frac{1}{3}-\frac{5c}{24}\right)\left(\frac{4}{3}-\frac{c}{3}\right)+c\left(\frac{1}{3}-\frac{5c}{24}\right)+\frac{c\left(1-c\right)}{2}+\frac{c^{2}}{4}\geq 0\text{,}\\ \text{Tr}\left(W_{4}⊗W_{4}⊗W_{4}\rho_{c}^{⊗2}\right)=c\left(\frac{4}{3}-\frac{c}{3}\right)\geq 0\text{,} \end{array}$$

With respect to the calculation of $S_{7}=\text{Tr}\left(\left(W_{4,AB^{\prime }}⊗W_{3,BC^{\prime }}⊗W_{3,CA^{\prime }}\right)\rho_{c}^{⊗2}\right)$, measurement results of two copies of tripartite state $\rho_{c}$ using bipartite witness operators $W_{3}$ and $W_{4}$ based on our nonlinear strategy, we only list the terms that contribute to the trace for simplicity,

$$\begin{array}{l} |000010\rangle \langle 000010|\overset{W_{4,AB^{\prime }}}{\underset{W_{3,BC^{\prime }},W_{3,CA^{\prime }}}{\to }}2|000010\rangle \langle 000010|-|100000\rangle \langle 000010|\text{,}\\ |001010\rangle \langle 100100|\overset{W_{4,AB^{\prime }}}{\underset{W_{3,BC^{\prime }},W_{3,CA^{\prime }}}{\to }}2|000110\rangle \langle 100100|-|100100\rangle \langle 100100|\text{,}\\ |001110\rangle \langle 001110|\overset{W_{4,AB^{\prime }}}{\underset{W_{3,BC^{\prime }},W_{3,CA^{\prime }}}{\to }}2|001110\rangle \langle 001110|-|101100\rangle \langle 001110|\text{,}\\ |010010\rangle \langle 100001|\overset{W_{4,AB^{\prime }}}{\underset{W_{3,BC^{\prime }},W_{3,CA^{\prime }}}{\to }}2|000011\rangle \langle 100001|-|100001\rangle \langle 100001|\text{,}\\ |010011\rangle \langle 010011|\overset{W_{4,AB^{\prime }}}{\underset{W_{3,BC^{\prime }},W_{3,CA^{\prime }}}{\to }}2|010011\rangle \langle 010011|-|110001\rangle \langle 010011|\text{,}\\ |011111\rangle \langle 011111|\overset{W_{4,AB^{\prime }}}{\underset{W_{3,BC^{\prime }},W_{3,CA^{\prime }}}{\to }}2|011111\rangle \langle 011111|-|111101\rangle \langle 011111|\text{,}\\ |100000\rangle \langle 100000|\overset{W_{4,AB^{\prime }}}{\underset{W_{3,BC^{\prime }},W_{3,CA^{\prime }}}{\to }}2|100000\rangle \langle 100000|-|000010\rangle \langle 100000|\text{,}\\ |100001\rangle \langle 010010|\overset{W_{4,AB^{\prime }}}{\underset{W_{3,BC^{\prime }},W_{3,CA^{\prime }}}{\to }}2|110000\rangle \langle 010010|-|010010\rangle \langle 010010|\text{,}\\ |100100\rangle \langle 001010|\overset{W_{4,AB^{\prime }}}{\underset{W_{3,BC^{\prime }},W_{3,CA^{\prime }}}{\to }}2|101000\rangle \langle 001010|-|001010\rangle \langle 001010|\text{,}\\ |101100\rangle \langle 101100|\overset{W_{4,AB^{\prime }}}{\underset{W_{3,BC^{\prime }},W_{3,CA^{\prime }}}{\to }}2|101100\rangle \langle 101100|-|001110\rangle \langle 101100|\text{,}\\ |110001\rangle \langle 110001|\overset{W_{4,AB^{\prime }}}{\underset{W_{3,BC^{\prime }},W_{3,CA^{\prime }}}{\to }}2|110001\rangle \langle 110001|-|010011\rangle \langle 110001|\text{,}\\ |111101\rangle \langle 111101|\overset{W_{4,AB^{\prime }}}{\underset{W_{3,BC^{\prime }},W_{3,CA^{\prime }}}{\to }}2|111101\rangle \langle 111101|-|011111\rangle \langle 111101|\text{.} \end{array}$$

Hence, we have

$$S_{7}=\text{Tr}\left(\left(W_{4,AB^{\prime }}⊗W_{3,BC^{\prime }}⊗W_{3,CA^{\prime }}\right)\rho_{c}^{⊗2}\right)=2.{\left(\frac{c}{8}\right)}^{2}+12.\frac{c}{8}.\frac{8-5c}{24}+4.{\left(\frac{1-c}{3}\right)}^{2}\text{,}$$

where $S_{7}<0$ when c<0.406, related to Figure 5.

#### The details of entanglement concentration

We compute the reduced state associated with the subsystem $AB^{\prime }$ after measurement. For clarity and intuitiveness, we adopt the representation method shown in Figure 1, where the first factor contains the measurement each party performs and the second factor contains the copies of the state. We have

$$\xi =\frac{1}{p_{m}}\frac{1}{d^{3}}\sum_{i,j,k,l,r,s=0}^{d-1}{\text{Tr}}_{BA^{\prime }}\left(\left(\begin{array}{cc} 1 & 1\\ {\left(\Psi^{†}\right)}^{-1}|i\rangle \langle j| & |i\rangle \langle j|{\left(\Psi^{T}\right)}^{-1} \end{array}\right)\left(\begin{array}{cc} \Psi^{T}|k\rangle \langle l| & |r\rangle \langle s|\Psi^{†}\\ |k\rangle \langle l|\Psi^{†} & \Psi^{T}|r\rangle \langle s| \end{array}\right)\right)=\frac{1}{p_{m}}\frac{1}{d^{3}}\sum_{i,j,k,l,r,s=0}^{d-1}\left(\begin{array}{cc} \Psi^{T}|k\rangle \langle l| & |r\rangle \langle s|\Psi^{†}\\ \langle j|k\rangle \langle l|i\rangle & \langle j|r\rangle \langle s|i\rangle \end{array}\right)=\frac{1}{p_{m}}\frac{1}{d^{3}}\sum_{k,l=0}^{d-1}\Psi^{T}|k\rangle \langle l|⊗|k\rangle \langle l|\Psi^{†}=\frac{1}{p_{m}}\frac{1}{d^{2}}|\varphi \rangle \langle \varphi |\text{,}$$

where $p_{m}=\text{Tr}\left(1_{AB^{\prime }}⊗|m\rangle {\langle m|}_{BA^{\prime }}|\varphi \rangle {\langle \varphi |}^{⊗2}\right)$, which means that the final state on $AB^{\prime }$ is |φ〉 with probability $\frac{1}{d^{2}p_{m}}$.

In the above scenario, if we replace the measurement acting on $BA^{\prime }$ with |M〉〈M|, where

$$|M\rangle =1⊗{\left(\Psi^{*}\Psi^{*}\right)}^{-1}|\psi^{+}\rangle ={\left(\Psi^{†}\right)}^{-1}⊗{\left(\Psi^{*}\right)}^{-1}|\psi^{+}\rangle \text{,}$$

then by calculating the reduced state associated with subsystem $AB^{\prime }$ after measurement, we obtain

$$\xi^{\prime }=\frac{1}{p_{m}^{\prime }}\frac{1}{d^{3}}\sum_{i,j,k,l,r,s=0}^{d-1}{\text{Tr}}_{BA^{\prime }}\left(\left(\begin{array}{cc} 1 & 1\\ {\left(\Psi^{†}\right)}^{-1}|i\rangle \langle j|\Psi^{-1} & {\left(\Psi^{*}\right)}^{-1}|i\rangle \langle j|{\left(\Psi^{T}\right)}^{-1} \end{array}\right)\left(\begin{array}{cc} |k\rangle \langle l| & |r\rangle \langle s|\\ \Psi |k\rangle \langle l|\Psi^{†} & \Psi^{T}|r\rangle \langle s|\Psi^{*} \end{array}\right)\right)=\frac{1}{p_{m}^{\prime }}\frac{1}{d^{3}}\sum_{i,j,k,l,r,s=0}^{d-1}\left(\begin{array}{cc} |k\rangle \langle l| & |r\rangle \langle s|\\ \langle j|k\rangle \langle l|i\rangle & \langle j|r\rangle \langle s|i\rangle \end{array}\right)=\frac{1}{p_{m}^{\prime }}\frac{1}{d^{3}}\sum_{k,l=0}^{d-1}|k\rangle \langle l|⊗|k\rangle \langle l|=\frac{1}{p_{m}^{\prime }}\frac{1}{d^{2}}|\psi^{+}\rangle \langle \psi^{+}|\text{,}$$

with $p_{m}^{\prime }=\text{Tr}\left(1_{AB^{\prime }}⊗|M\rangle {\langle M|}_{BA^{\prime }}|\varphi \rangle {\langle \varphi |}^{⊗2}\right)$. This means that the final state in $AB^{\prime }$ becomes a maximally entangled one with a positive probability.

### Quantification and statistical analysis

Statistical analysis was not used in this work.
